# Policies and interventions addressing child undernutrition in Latin America and the Caribbean: a scoping review

**DOI:** 10.3389/fpubh.2026.1871789

**Published:** 2026-09-23

**Authors:** Jesús Endara-Mina, Jhon Paul Guerrero-González, Cristopher-Jordán Osorio, Cristopher-Josué Escudero, Dario Chicaiza, Katherine Andrade-Trávez, Brenda Tejada-Grijalva, Kevin Padilla-Olivo, Carlos Astudillo-Abad, Robinson Arrobo-Camacho, Sandra Luzuriaga-Morejón, María Belén Mena-Ayala

**Affiliations:** 1 Facultad de Ciencias Jurídicas y Políticas, Universidad Técnica Particular de Loja (UTPL), Loja, Ecuador; 2 Coordinación de Docencia e Investigación, Hospital Provincial General Pablo Arturo Suárez, Quito, Ecuador; 3 Centro de Investigación Para La Salud en América Latina (CISeAL), Facultad de Medicina, Pontificia Universidad Católica del Ecuador (PUCE), Quito, Ecuador; 4 Asociación Científica de Estudiantes de Medicina, Universidad Central del Ecuador, Quito, Ecuador; 5 Hospital General del Sur de Quito, Instituto Ecuatoriano de Seguridad Social, Quito, Ecuador; 6 Facultad de Ciencias Médicas, Universidad Central del Ecuador (UCE), Quito, Ecuador; 7 Facultad de Medicina, Universidad de las Américas (UDLA), Quito, Ecuador; 8 Hospital Básico de Paute, Ministerio de Salud Pública del Ecuador, Paute, Ecuador; 9 Servicio de Pediatría y Neonatología, Hospital Provincial General Pablo Arturo Suárez, Quito, Ecuador; 10 Facultad de Ciencias Médicas Universidad Central del Ecuador, Instituto de Biomedicina, Quito, Ecuador; 11 Facultad de Medicina, Pontificia Universidad Católica del Ecuador (PUCE), Quito, Ecuador

**Keywords:** child undernutrition, Latin America and the Caribbean, nutrition programs, public policy, social protection

## Abstract

**Background/objectives:**

Child undernutrition remains a major public health issue in Latin America and the Caribbean (LAC), driven by structural inequalities. This study aimed to synthesize evidence on policies, programs, and interventions addressing child undernutrition in the region.

**Methods:**

A scoping review was conducted following the Joanna Briggs Institute methodology and reported according to PRISMA-ScR guidelines. A comprehensive search was performed in PubMed/MEDLINE, Scopus, Web of Science, Dimensions, SciELO, and the Virtual Health Library without language or time restrictions. Studies evaluating or describing implemented policies, programs, or interventions targeting undernutrition in children.

**Results:**

A total of 81 studies were analyzed. Most focused on national-level interventions using quantitative designs. Nutrition-sensitive strategies, particularly cash transfer programs, were most frequently evaluated. Stunting was the main outcome. Findings were heterogeneous: about one-third reported reductions in undernutrition, while most showed mixed or limited effects.

**Conclusions:**

Although policies in LAC have evolved toward multisectoral approaches, their impact remains uneven. Strengthening governance, coordination, and implementation is key to achieving sustained reductions in child undernutrition.

## 1 Introduction

Child undernutrition continues to represent one of the most persistent and com-plex challenges for global public health. Despite the progress observed in recent decades, it is estimated that in 2024, stunting or chronic undernutrition affected 23.2% of children under five years of age worldwide, corresponding to approximately 150.2 million children, while acute undernutrition or wasting threatened the lives of around 6.6%, equivalent to 42.8 million children (1–4).

Child undernutrition primarily originates from inadequate intake of essential nutrients, recurrent infections, and adverse structural conditions, and manifests in various clinical forms, including stunting, acute malnutrition, and, paradoxically, childhood overweight and obesity (5, 6). These forms of malnutrition are not mutually exclusive and may coexist within the same country, community, or even household, giving rise to what is known as the double or triple burden of malnutrition (7). The consequences of undernutrition in early childhood are profound and long-lasting, affecting physical and cognitive development, learning capacity, school performance, and economic productivity in adulthood, thereby perpetuating intergenerational cycles of poverty and inequality (8–11).

Child undernutrition also imposes substantial costs on health systems and economies in Latin America and the Caribbean (LAC). In the region, it has been estimated that the economic cost of stunting may translate into a per capita income approximately 5% lower compared to a scenario without chronic undernutrition (12). Furthermore, World Bank reports indicate that, in many countries, undernutrition may lead to losses on the order of 2–3% of gross domestic product (GDP) due to reduced productivity and human capital (13). In LAC, studies by ECLAC on the cost of hunger have documented that child undernutrition represents a significant economic burden, for instance, 3.3% of the combined GDP in Bolivia, Ecuador, Paraguay, and Peru in 2005, with particularly high impacts in some countries, such as Guatemala, where the combined cost of mal-nutrition reached 16.3% of GDP in 2018 (14, 15). Additionally, undernourished children incur higher direct healthcare costs during childhood, increasing financial pressure on health systems and limiting the sustainability of public policies.

Scientific evidence has demonstrated that the first 1,000 days of life, from conception to two years of age, constitute a critical period during which exposure to nutritional deficits can lead to irreversible damage in neurological, metabolic, and immunological development (16, 17). Undernutrition during this early window is associated with reduced cognitive development, increased susceptibility to infections, and a higher risk of chronic non-communicable diseases later in life, contributing to the persistence of health and well-being disadvantages across the life course, particularly in contexts of high social vulnerability (18–20).

In LAC, child undernutrition remains a major cause of preventable morbidity and mortality. It is estimated that approximately 152,000 children died in the region in 2022 from causes related to nutritional deficiencies, more than half of whom were neonates under 28 days of age (21). Although the region has experienced a sustained decline in chronic child undernutrition since the late 21th century, this progress has been uneven, with marked disparities between and within countries.

The prevalence of child undernutrition in LAC is closely linked to structural social determinants, such as multidimensional poverty, food insecurity, limited access to health and sanitation services, and profound ethnic, territorial, and educational gaps. Numerous studies have documented that undernutrition is significantly more prevalent among Indigenous and Afro-descendant populations, rural communities, and households with low maternal educational attainment (1, 22–24). These inequalities reflect the interaction of economic, social, cultural, and political factors that shape access to adequate diets and essential services.

In response to this situation, LAC countries have implemented a wide range of interventions and public policies, including complementary and school feeding programs, conditional cash transfers, primary health care strategies, social protection programs, food fortification, and intersectoral approaches aimed at improving child nutrition (5–9, 22–26). However, the available evidence regarding their effectiveness is heterogeneous, fragmented, and evaluated under diverse methodological frameworks, ranging from community trials and quasi-experimental studies to descriptive analyses, prospective modeling, and implementation studies.

The lack of a comprehensive synthesis of this evidence hinders the identification of regional patterns, the comparison of strategies, and the understanding of the contexts in which certain interventions have proven more effective. In this regard, the present study aims to map and synthesize the available evidence on policies, programs, and interventions addressing child undernutrition in LAC.

## 2 Material and methods

This study was conducted following the Joanna Briggs Institute (JBI) methodology for scoping reviews (27) and is reported in accordance with the Preferred Reporting Items for Systematic Reviews and Meta-Analyses extension for Scoping Reviews (PRISMA-ScR) guidelines (Supplementary Table S1) (28).

### 2.1 Definitions

Child undernutrition: Child undernutrition was defined as a condition resulting from inadequate intake or absorption of essential nutrients, commonly expressed as stunting (low height-for-age), wasting (low weight-for-height), and underweight (low weight-for-age) in children under five years of age. Studies reporting composite indicators of child undernutrition were also considered.

Policies, programs, and interventions: For the purposes of this review, policies, programs, or interventions were defined as structured public actions implemented at national or subnational level, including but not limited to nutrition-specific interventions (e.g., supplementary feeding, food fortification), nutrition-sensitive strategies (e.g., cash transfer programs, school feeding programs), and multisectoral public policies explicitly aimed at reducing child undernutrition.

Geographic scope: Latin America and the Caribbean were defined according to standard international classifications, including Central America, South America, Mexico, and Caribbean sovereign states.

### 2.2 Search strategy

The strategy combined Medical Subject Headings (MeSH) and free-text terms covering child undernutrition (e.g., stunting, wasting, underweight), nutrition-related interventions, public and social policies, and the geographic scope of the region (Table 1). Boolean operators were used to maximize retrieval and ensure inclusion of heterogeneous study designs commonly used in policy and program evaluation research. To ensure broad coverage of the evidence base, no restrictions were applied with respect to language or publication period, allowing the inclusion of studies published in Spanish and Portuguese as well as earlier evaluations of long-standing policies and programs. All references were exported to Rayyan Web (29), where duplicates were identified and removed prior to screening.

**Table 1:** Summary of the search strategy.

Concept	MeSH terms	Free terms/natural language	Boolean combination
Child undernutrition	“Child Nutrition Disorders”[MeSH] OR “Malnutrition”[MeSH] OR “Undernutrition”[MeSH] OR “Growth Disorders”[MeSH]	child undernutrition OR child malnutrition OR stunting OR wasting OR underweight OR growth faltering OR chronic malnutrition	(Child Nutrition Disorders OR Malnutrition OR Undernutrition OR Stunting OR Wasting)
Population	“Infant”[MeSH] OR “Child, Preschool”[MeSH] OR “Child”[MeSH]	infant^*^ OR child^*^ OR preschool child^*^ OR children under five OR under-5 child^*^	(Infant OR Child OR Preschool)
Policies/programs/interventions	“Public Policy”[MeSH] OR “Health Policy”[MeSH] OR “Nutrition Programs”[MeSH] OR “Food Assistance”[MeSH] OR “School Feeding Programs”[MeSH]	policy OR program^*^ OR intervention^*^ OR cash transfer^*^ OR school feeding OR food supplementation OR nutrition intervention^*^ OR Social protection	(Policy OR Program^*^ OR Intervention^*^ OR Nutrition Program^*^)
Programmatic approaches	“Social Protection”[MeSH] OR “Primary Health Care”[MeSH] OR “Community Health Services”[MeSH]	conditional cash transfer^*^ OR primary health care OR community-based program^*^ OR maternal and child health OR multisectoral intervention^*^	(Social Protection OR Primary Health Care OR Community-based)
Geographic scope	“Latin America”[MeSH] OR “Caribbean Region”[MeSH]	Latin America OR Caribbean OR Argentina OR Bolivia OR Brazil OR Chile OR Colombia OR Costa Rica OR Cuba OR Dominican Republic OR Ecuador OR El Salvador OR Guatemala OR Haiti OR Honduras OR Jamaica OR Mexico OR Nicaragua OR Panama OR Paraguay OR Peru OR Uruguay OR Venezuela OR Belize OR Bahamas OR Barbados OR Dominica OR Grenada OR Guyana OR Saint Lucia OR Saint Kitts and Nevis OR Saint Vincent and the Grenadines OR Suriname OR Trinidad and Tobago	(Latin America OR Caribbean OR Country-specific terms)

MeSH, Medical Subject Headings.

### 2.3 Information sources

The electronic search was conducted across multiple international and regional bibliographic databases to capture both global and region-specific evidence. These included PubMed/MEDLINE, Web of Science, Scopus, Dimensions, the Virtual Health Library and SciELO.

### 2.4 Study selection

In the first stage, two reviewers (A-TK and OC-J) independently screened titles and abstracts. Discrepancies were resolved through discussion or by a third reviewer (E-MJ). In the second stage, full texts were assessed in detail following the same procedure, and the study selection process is summarized in a PRISMA-ScR flow diagram (Figure 1).

**Figure 1:**
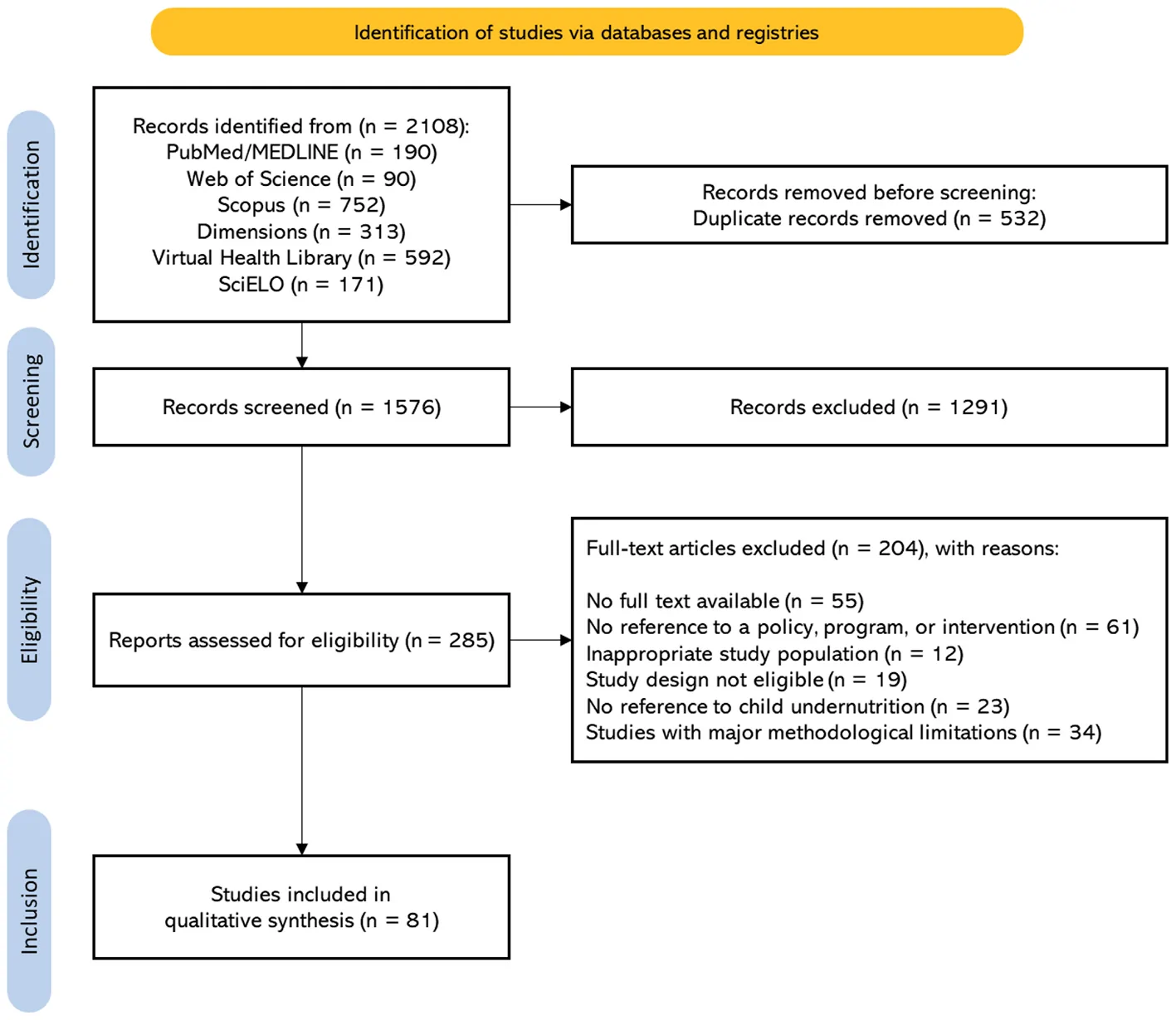
PRISMA flow diagram of the study selection process.

Inclusion criteria encompassed studies evaluating or describing implemented public policies, programs, or interventions aimed at preventing or reducing child undernutrition among children under five years of age, including outcomes such as stunting, wasting, underweight, or other anthropometric indicators.

Studies employing quantitative, qualitative, mixed-methods, quasi-experimental, and experimental designs were considered eligible. In addition, narrative reviews, systematic reviews, scoping reviews, multicountry case series, and programmatic reports were included when they provided relevant evidence on implemented policies, programs, or interventions addressing child undernutrition in the region, particularly when such designs were appropriate for capturing policy implementation, coverage, or contextual effects.

Exclusion criteria comprised editorials, commentaries, letters to the editor, pre-prints, opinion pieces, conference abstracts, animal studies, and publications without full-text access. Individual clinical case reports without a programmatic or policy component were also excluded. Studies focusing exclusively on biological mechanisms or clinical management at the individual level, without reference to an implemented policy, program, or population-level intervention, were not considered eligible.

### 2.5 Data extraction and management

Two reviewers (G-GJ and E-MJ) developed a data extraction form refined by the author team. Data were independently extracted by reviewers (OC-J, EC-J, A-TK, CD and T-GB), and discrepancies were resolved by a reviewer (E-MJ).

For each included study, the following variables were systematically extracted: author(s) and year of publication, country or countries evaluated, type and name of the policy, program, or intervention, study design, target population and age group, analytical approach, nutrition-related outcomes assessed, and main findings, including the direction and magnitude of reported effects. Extracted data were organized into structured tables to facilitate descriptive synthesis, comparison across countries, and identification of thematic patterns and evidence gaps within the region.

### 2.6 Data analysis and synthesis

The included studies were analyzed using a descriptive and narrative synthesis approach, consistent with the objectives of a scoping review and the heterogeneity of the available evidence. Analysis focused on study design, geographic scope, target populations, type of policy, program, or intervention, and reported nutrition-related outcomes, with particular attention to how interventions were conceptualized, implemented, and evaluated across different country contexts.

To facilitate interpretation, policies, programs, and interventions were grouped thematically according to sector and intervention type, including but not limited to nutrition-specific interventions (e.g., supplementary feeding, food fortification), nutrition-sensitive strategies (e.g., cash transfer programs, school feeding programs), and multisectoral or governance-oriented policies integrating health, social protection, education, and food systems. This classification allowed for the identification of recurrent patterns, dominant policy approaches, and gaps in implementation across the region.

In addition to thematic grouping, findings were synthesized at the country level, enabling a descriptive mapping of the policies and programs implemented in each Latin American and Caribbean country represented in the final sample. This approach supported comparison across countries and highlighted heterogeneity in policy intensity, intervention portfolios, and evaluative evidence, rather than attempting to pool effect estimates or draw causal inferences.

## 3 Results

The initial search identified a total of 2108 publications. After removing duplicates, 1,576 studies were eligible for title and abstract screening. Subsequently, 285 full-text studies were reviewed, and 81 studies met the inclusion criteria. The selection process adhered to the previously established eligibility criteria. A summary of the bibliographic search and study selection is provided in Figure 1.

### 3.1 Characteristics of the included studies

Table 2 summarizes the general characteristics of the 81 studies included in this scoping review, categorized according to study design, level and sector of intervention, target population, and reported nutrition-related outcomes. Most studies employed quantitative methodologies, with a predominance of observational analytical designs and quasi-experimental evaluations, while a smaller proportion consisted of randomized or cluster-randomized trials. Qualitative and mixed-methods approaches were less frequent and were primarily used to examine implementation processes, governance, and contextual factors influencing program performance.

**Table 2:** Summary of study characteristics.

Characteristic	Category	*n* (%)
Study design	Reviews	18 (22.2)
	Randomized or cluster-randomized trials	6 (7.4)
	Quasi-experimental studies	22 (27.2)
	Observational analytical studies	25 (30.9)
	Qualitative or mixed-methods studies	10 (12.3)
Level of intervention	National-level policies or programs	59 (72.8)
	Subnational or municipal programs	12 (14.8)
	Community-based interventions	10 (12.3)
Sectoral approach	Nutrition-specific interventions	21 (25.9)
	Nutrition-sensitive interventions	33 (40.7)
	Education–health integrated interventions	15 (18.5)
	Multisectoral or governance-based strategies	12 (14.8)
Target population^*^	Children under five years of age	81 (100)
	First 1,000 days (0–23 months)	47 (58.0)
	School-aged children (≥5 years)	21 (25.9)
Primary outcomes assessed^*^	Stunting (height-for-age)	65 (80.2)
	Wasting (weight-for-height)	29 (35.8)
	Underweight (weight-for-age)	25 (30.9)
	Intermediate outcomes (dietary diversity, service utilization, coverage)	41 (50.6)
Direction of reported effects	Reduction in undernutrition indicators	30 (37.0)
	Mixed effects	40 (49.4)
	No effect	11 (13.6)
Geographic scope	Single-country studies	64 (79.0)
	Multicountry or regional studies	17 (21.0)

^*^The sum of frequencies across categories may exceed the total number of included studies (*n* = 81), as individual studies could be classified into more than one category (e.g., multiple intervention types, target populations, or outcomes).

The study populations focused predominantly on children under five years of age, with more than half of the studies explicitly targeting the first 1,000 days of life (0–23 months). In contrast, a smaller subset included school-aged children, mainly in evaluations of school feeding or education/health integrated interventions. Most evaluations assessed national-level policies or programs, although subnational, municipal, and community-based interventions were also represented.

Regarding intervention types, the evidence was largely concentrated on nutrition-sensitive strategies, particularly social protection and cash transfer programs, followed by nutrition-specific interventions such as supplementary feeding and food-assisted maternal and child health programs. Education–health integrated initiatives and multisectoral or governance-based strategies were less frequently evaluated. Across studies, stunting was the most reported outcome, followed by wasting, underweight, and intermediate indicators such as dietary diversity and service utilization.

Overall, reported effects were heterogeneous, with just over one-third of studies documenting reductions in undernutrition indicators, while the majority reported mixed effects and a smaller proportion found no measurable impact. Taken together, these findings highlight a literature dominated by quantitative program evaluations, characterized by substantial heterogeneity in intervention types and outcomes, and a comparatively limited focus on implementation and contextual dynamics across countries in Latin America and the Caribbean.

### 3.2 Overview of policy and programmatic approaches to address child undernutrition in Latin America and the Caribbean

The 81 studies included in this scoping review provide a comprehensive overview of the diverse policy and programmatic approaches implemented to address child undernutrition across Latin America and the Caribbean. Collectively, these studies encompass national and subnational policy analyses, program evaluations, implementation studies, and mixed-methods research conducted over several decades and across a wide range of country contexts, including Chile, Brazil, Mexico, Peru, Colombia, Ecuador, Argentina, Paraguay, Venezuela, Bolivia, Guatemala, Haiti, and multiple Central American and Caribbean settings (30**?** –93).

Across this body of evidence, policy responses to child undernutrition show a clear temporal evolution. Earlier studies primarily document nutrition-specific interventions, including food supplementation, food fortification, growth monitoring, and targeted feeding programs delivered through the health sector or social assistance schemes (30, 33–35, 37, 40). Over time, the focus expanded toward broader nutrition-sensitive approaches embedded within social protection policies, food and nutrition security strategies, early childhood development frameworks, and poverty reduction programs (49, 50, 53, 54, 66, 77, 89).

The studies consistently conceptualize child undernutrition, particularly chronic undernutrition and stunting, as a multidimensional outcome shaped by socioeconomic inequality, household food insecurity, maternal education, access to health services, sanitation, and territorial disparities. Evidence from Brazil, Mexico, Peru, Ecuador, Colombia, and Venezuela highlights that isolated nutrition interventions yield limited and heterogeneous effects when structural determinants are not simultaneously addressed (43, 51, 60, 62, 64, 73, 81).

Considerable heterogeneity in policy design, implementation capacity, and effectiveness is reported across countries and governance levels. While several studies document improvements in intermediate outcomes, such as dietary diversity, breast-feeding practices, and service utilization, others report modest or null impacts on stunting reduction, particularly in rural areas, indigenous populations, and contexts characterized by institutional fragility or political instability (46, 55, 63, 65, 68–70).

Importantly, the collective evidence frames child undernutrition not merely as a technical or health-sector issue, but as a governance and development challenge. Mul-tiple studies emphasize the importance of political commitment, legal frameworks, multisectoral coordination, stable financing, and monitoring systems in shaping program sustainability and equity (49, 50, 66, 84). Conversely, fragmented governance arrangements, short-term policy cycles, and weak institutional capacity are repeatedly identified as barriers to sustained impact across the region (67, 79, 86).

Overall, the synthesis of the 81 studies demonstrates that Latin America and the Caribbean have developed a diverse and increasingly comprehensive portfolio of policy and programmatic responses to child undernutrition. However, persistent structural inequities, uneven implementation, and governance constraints continue to limit the translation of policy commitments into sustained and equitable reductions in child undernutrition across the region (77, 78, 85, 88).

### 3.3 National-level policies and programs

National-level policies and programs addressing child undernutrition across Latin America and the Caribbean are extensively documented in the included studies, which describe a wide range of nutrition-specific and nutrition-sensitive interventions implemented through health systems, social protection mechanisms, food assistance schemes, and national food and nutrition security strategies. Across countries, these policies have evolved over time from targeted food supplementation initiatives toward more integrated approaches that combine health, education, social protection, and food system interventions, although the scope, maturity, and effectiveness of national responses vary substantially by context.

#### 3.3.1 Peru

Evidence from Peru highlights a progressive consolidation of national strategies combining food assistance, early childhood development, and social protection. Multiple studies describe the implementation and evolution of programs such as Programa Vaso de Leche and Qali Warma, which provide food supplementation to children through schools and community mechanisms, as well as multisectoral strategies such as CRECER, designed to reduce chronic child undernutrition through coordinated actions across health, education, sanitation, and social inclusion sectors (42, 53, 90, 94). Additional national-level interventions include conditional cash transfer programs (JUNTOS), early childhood services (Cuna Más), and integrated nutrition actions evaluated in both policy analyses and empirical studies (59, 68, 77, 89).

#### 3.3.2 Chile

The Chilean experience is characterized by long-standing national nutrition policies with a strong institutional foundation. Several studies document the historical role of complementary feeding programs such as the Programa Nacional de Alimentación Complementaria (PNAC), including the distribution of fortified products such as Leche Purita, which have been associated with improvements in child nutritional status and micronutrient intake over several decades (16, 35, 70). More recent evidence highlights programmatic adaptations within PNAC, including changes in product composition and fortification strategies, as well as broader regulatory public health measures such as front-of-package food labeling and restrictions on food marketing to children (74, 78, 88).

#### 3.3.3 Paraguay

National-level programs in Paraguay are primarily described through food supplementation and integrated nutrition support initiatives. Studies document the implementation of the Programa Alimentario Nutricional Integral (PANI) and related strategies aimed at reducing undernutrition among vulnerable children, while also re-porting challenges related to program adherence, coverage, and continuity (32, 46, 79).

#### 3.3.4 Brazil

Brazil presents the most extensive body of national-level evidence among the included studies. Multiple analyses describe the role of large-scale social protection pro-grams, most notably Bolsa Família, in improving child nutrition outcomes through income support linked to health and education conditionalities (23, 47, 48, 50). These programs are frequently discussed within the broader framework of national food and nutrition security strategies, including Fome Zero, which integrated income redistribution, food access, health services, and community participation (82, 95). Additional studies examine specific national nutrition actions, health system–based interventions, and governance arrangements shaping policy implementation and equity (60, 65, 72, 73, 82, 83, 86, 91–93, 96**?** –100).

#### 3.3.5 Mexico

National policies in Mexico are documented through a range of food assistance, social protection, and nutrition programs implemented over different periods. Studies describe initiatives such as community kitchens (Comedores Comunitarios), targeted food subsidies (including tortilla subsidy schemes), and large-scale national strategies aimed at reducing hunger and poverty (34, 101, 102). Additional analyses focus on national nutrition programs targeting children and families, including school-based and community-based interventions, as well as broader policy frameworks integrating health, education, and food access (36, 37, 58).

#### 3.3.6 Colombia

National-level approaches in Colombia are described through both historical planning instruments and large-scale social protection programs linked to nutrition. Several studies document the role of conditional cash transfers, particularly Familias en Acción, and their relationship with child nutrition and household food security out-comes, framed within broader national strategies aimed at reducing poverty and improving human capital (51, 57). Additional evidence discusses national policy cycles and planning approaches for food and nutrition, including analyses of national guidance instruments and programmatic strategies that integrate health and nutrition actions at scale (32, 39, 56, 81).

#### 3.3.7 Ecuador

National-level evidence from Ecuador describes a combination of social protection policies, food and nutrition programs, and health system-linked interventions aimed at addressing child undernutrition. While cash transfer schemes constitute a central component of the national response, the evidence base also documents complementary programmatic actions embedded within food assistance, nutrition surveillance, and health service delivery.

Several studies analyze the role of social protection instruments, particularly the Bono de Desarrollo Humano (BDH) and related transfer modalities, highlighting their association with nutrition-relevant behaviors and child health outcomes when combined with access to basic services (61, 62). These programs are framed as part of Ecuador's broader poverty reduction strategy and are frequently discussed in relation to their conditionalities or expected links to health and nutrition service utilization.

Beyond cash transfers, national evidence also documents food-based and nutrition-specific programs, including public food assistance mechanisms and nutrition actions coordinated through the health system. Studies describe the implementation of national strategies oriented toward improving child nutrition outcomes through integrated maternal and child health services, growth monitoring, and nutrition counseling delivered via the public health network (80). These interventions are contextualized within Ecuador's efforts to strengthen primary health care and address persistent inequalities affecting rural, Indigenous, and low-income populations.

Ecuador additionally appears in comparative and multi-country analyses examining national policy mixes and governance arrangements for child undernutrition. These studies situate Ecuador's experience alongside other Andean and Latin American countries, emphasizing common challenges related to intersectoral coordination, policy continuity, and the alignment of social protection, food security, and health system interventions (54, 76, 103). Collectively, this body of evidence suggests that Ecuador's national response to child undernutrition relies on a hybrid model combining income support, food-based programs, and health sector actions, with effectiveness contingent on the degree of integration across sectors and levels of governance.

#### 3.3.8 Argentina

Argentina's national policy response described in the included studies centers on food assistance and school feeding mechanisms (e.g., school canteens/comedores), national social protection measures, and more recent anti-hunger strategies. Studies discuss national-level food support and institutional arrangements shaping access to nutrition among children and low-income households (38, 49). Pandemic-era evidence and recent analyses also reference national packages and policy orientations aimed at mitigating food insecurity and protecting child nutrition, including broader national anti-hunger initiatives and the role of macroeconomic conditions in shaping nutrition outcomes (64, 85, 104).

#### 3.3.9 Venezuela

National-level approaches in Venezuela are described primarily through food distribution and food security programs, governance narratives, and rights-based frames, often in the context of political and economic crisis. Several studies describe national policy responses characterized by centralized food distribution schemes (including CLAP-related mechanisms) and broader debates about the right to food, institutional capacity, and governance (52, 67). Additional studies examine nutrition challenges and policy responses affecting children and households, including programmatic and structural determinants of food insecurity and nutrition-related vulnerabilities (69–71, 75).

#### 3.3.10 Guatemala

Evidence from Guatemala reflects a national policy mix combining program delivery models and food-system instruments. One set of studies focuses on community-based maternal–child interventions designed to improve diet quality and reduce undernutrition, such as PROCOMIDA and related programmatic approaches (66). Complementary evidence describes national-level strategies focused on the fortification of staple foods (e.g., wheat flour and sugar, and modeled fortification scenarios), framing fortification as a key national instrument to reduce micronutrient deficiencies and improve child nutrition at scale (44).

#### 3.3.11 Haiti

National-level approaches in Haiti are represented in the included studies through analyses of maternal–child nutrition programs and infant feeding practices, often situated within contexts of structural vulnerability and constrained health system capacity. Evidence includes policy-relevant work aligned with WHO recommendations on feeding practices and programmatic actions targeting child nutrition and maternal health (45, 55). Additional studies consider determinants and outcomes relevant to national program effectiveness, including nutrition-related vulnerabilities and policy implications for supporting infants and young children (63).

#### 3.3.12 Bolivia

Bolivia appears in the included evidence both through single-country and cross-country studies documenting national programmatic responses. National-level approaches include food transfers and policy actions aimed at reducing child undernutrition, and Bolivia is also included in comparative analyses of program modalities and national policy mixes across Andean and multi-country settings (54, 103, 105, 106).

#### 3.3.13 Cuba

One study provides a national framing of nutrition and health outcomes through Cuba's community-oriented and preventive health system model, positioning national health system organization as a key contextual determinant for child and population nutrition outcomes (31).

### 3.4 Education–health integrated interventions

Education–health integrated interventions emerge across the included studies as a key programmatic approach to address child undernutrition in Latin America and the Caribbean, particularly during early childhood and school-age periods. These interventions are characterized by the deliberate linkage of nutrition and health actions with educational settings, leveraging schools, early childhood centers, and community education platforms as delivery points for nutrition-sensitive and nutrition-specific strategies.

Several studies document the role of school feeding and complementary feeding programs as central education/health integration mechanisms. Evidence from Chile, Peru, Brazil, Argentina, and Mexico shows that school-based food provision not only contributes to dietary intake and micronutrient adequacy but also serves as an entry point for health monitoring, nutrition education, and referral to health services (30, 35, 36, 64, 94, 101). These programs are frequently embedded within national education systems, with health sector participation ensuring nutritional standards, growth monitoring, and food safety.

In Peru and Ecuador, education/health integration is also described through early childhood development platforms and community-based education initiatives that combine caregiver education, nutrition counseling, and health service linkage. Studies highlight how interventions implemented through childcare centers, early education programs, and community workshops aim to improve feeding practices, maternal knowledge, and timely use of health services (59, 61, 80, 89).

Brazilian studies provide extensive evidence on education–health synergies, particularly through the integration of nutrition actions within primary health care and school environments. Interventions described include nutrition education, promotion of healthy diets, school-based health actions, and coordination between municipal education departments and health services (48, 60, 98**?**, 99). These studies underscore that educational settings offer sustained contact with children and families, enhancing the reach and continuity of nutrition-related interventions.

In Mexico and Colombia, education/health integrated interventions are described through community nutrition education, school-based programs, and intersectoral initiatives linking social services, education, and health. Evidence highlights that programs combining food provision with nutrition education and health follow-up tend to be more effective than standalone interventions, particularly in marginalized communities (39, 57, 58, 102). However, the studies also note challenges related to coordination across sectors and variability in implementation quality across territories.

Cross-country and comparative studies further reinforce the relevance of education/health integration as a regional strategy. Multi-country analyses describe common patterns in the use of schools and early education platforms to deliver nutrition interventions, while also emphasizing the importance of aligning curricula, food provision standards, and health system engagement to achieve sustained nutrition outcomes (33, 37, 40, 84).

## 4 Discussion

The present study made it possible to identify the available evidence on policies, programs, and interventions aimed at addressing child undernutrition in Latin America and the Caribbean. The findings indicate that regional interventions have evolved from predominantly nutrition-specific approaches focused on limited groups (such as supplementation and micronutrient fortification) toward more comprehensive strategies that integrate social protection policies, food security, public health, and early childhood development. This shift reflects a multifactorial understanding of child undernutrition, in which biological, social, economic, and environmental determinants interact (107, 108).

Global studies have demonstrated that the sustainable reduction of stunting requires simultaneous interventions targeting multiple determinants, including maternal nutrition, access to health services, sanitation, and poverty reduction (107, 108). The importance of maternal nutritional status is grounded in the hypothesis proposed by epidemiologist David Barker in the 1980s, which posited that nutritional conditions during intrauterine life determine the risk of chronic diseases in adulthood. He observed that neonates with low birth weight were more likely to develop hypertension, type 2 diabetes, and coronary heart disease later in life (109). This led to the concept of Early Metabolic Programming, a biological process that produces permanent changes in the organism during the first 1,000 days of life, potentially beginning with fetal undernutrition, which induces a thrifty metabolism that predisposes individuals to childhood obesity and metabolic syndrome (110). The scientific evolution of Barker's hypothesis into a broader framework gave rise to the Developmental Origins of Health and Disease (DOHaD) approach, which emphasizes the interaction between genes and environment, reinforcing optimal maternal and infant nutrition as one of the most powerful public health tools (111, 112). The Latin American chapter of DOHaD has spent more than a decade promoting research and translating this knowledge into public policy across the region, highlighting how economic and nutritional crises in countries such as Brazil, Chile, and Argentina have left epigenetic imprints on their populations (113).

Thus, undernutrition cannot be explained solely by dietary intake. Marmot (114) compellingly demonstrated that socioeconomic position determines health in a graded and systematic manner: the lower the social position, the worse the health outcomes, following a gradient that transcends national boundaries. Bayati et al. (115) confirmed that socioeconomic inequality is the central social determinant of nutritional deficiencies worldwide, disproportionately affecting countries with lower Human Development Index levels. Ouaijan et al. (116) further showed that unemployment, low educational attainment, and food insecurity are directly associated with higher probabilities of undernutrition. Desai et al. (117) demonstrated that ethnicity, socioeconomic status, and geographic location shape household food insecurity, with tangible clinical consequences for the most vulnerable children.

Among the most important findings is the central role of nutrition-sensitive policies, particularly conditional cash transfer (CCT) programs, which constitute one of the most extensively evaluated interventions in the region (116, 118). Landmark programs such as Bolsa Família in Brazil, Progresa/Oportunidades in Mexico, and Familias en Acción in Colombia have been widely studied for their impact on child health and poverty reduction (23, 34, 37, 39, 48, 51, 57, 58, 60, 72, 82, 96, 100, 103). Evidence indicates that these programs can improve access to food, increase the use of preventive health services, and strengthen childcare practices (119). For instance, evaluations of CCT programs in Colombia have shown improvements in the utilization of preventive services, dietary diversity, and maternal and childcare practices among beneficiary households (57, 120). Likewise, regional reviews indicate that these programs have contributed to improvements in several determinants of child nutrition in Latin America (121, 122).

At the population level, cash transfers have also demonstrated important effects on child health indicators. A longitudinal analysis conducted in municipalities across Brazil, Mexico, and Ecuador found that the expansion of CCT programs was associated with significant reductions in child mortality and in poverty-related diseases, including undernutrition, respiratory infections, and diarrhea (123). Indeed, it is estimated that these programs have contributed to preventing more than 700,000 child deaths in Latin America over the past two decades (122). In Ecuador, evaluations of the Bono de Desarrollo Humano program have also identified significant reductions in child mortality from poverty-related causes, along with increased utilization of preventive health services (80). Despite the documented benefits of social protection programs such as CCTs, a critical evidence gap remains regarding their potential unintended impacts on childhood overweight and obesity. Given the rapid epidemiological transition in Latin America and the Caribbean, and the high prevalence of the double burden of malnutrition, very few included studies systematically evaluated whether cash transfers or food distribution schemes inadvertently promote the consumption of ultra-processed, energy-dense foods. Evaluating how nutrition-sensitive policies influence both ends of the malnutrition spectrum is essential for designing double-duty actions across the region.

However, the results also indicate that the impact of many interventions on anthropometric indicators remains inconsistent. Some studies report improvements in intermediate determinants (such as food security or access to health services) without observing significant reductions in the prevalence of chronic undernutrition (124, 125). Recent evaluations of transfer programs in Latin America have identified limited effects on anthropometric indicators in certain contexts, suggesting that the magnitude of the benefit depends on factors such as the size of the transfers, program coverage, and the capacity of health systems to respond to the increased demand (126, 127). This evidence aligns with previous analyses indicating that stunting is a cumulative indicator reflecting prolonged exposure to adverse socioeconomic conditions and structural determinants. Among these, environmental health programs, such as access to water, sanitation, and solid waste management, must also be considered (128).

Another key aspect involves integrated interventions across the health, education, and social protection sectors, including school feeding programs and early childhood development strategies. These programs use the education system as a platform to implement nutrition-sensitive interventions, facilitating access to nutritious foods, nutrition education, and health services (129–131). In Latin America, school feeding programs are among the most widespread social policies and have been associated with improvements in dietary intake, academic performance, and household food security among vulnerable populations (124, 132). Nevertheless, their nutritional impact largely depends on factors such as the quality of the food provided, the continuity of funding, and effective implementation at the local level (133, 134).

In countries that have achieved sustained success, community-based and primary health care interventions have laid the foundation for broader state programs. For ex-ample, in Chile, alongside community actions promoting breastfeeding, additional initiatives were implemented, such as the prevention of spina bifida and anencephaly through folic acid fortification of bread, which, together with other strategies, contributed to the development of the national program Chile Crece Contigo (74, 76, 78, 88). In Ecuador, community-adapted interventions such as the Lulun Project, a strategy de-signed to promote behavioral change toward egg consumption and its early introduction into complementary feeding, have demonstrated the effectiveness of social marketing techniques grounded in cultural norms and local values (135). Other interventions that have been implemented and may be considered include food labeling policies, which have already had measurable impacts in Ecuador and other Latin American countries (136, 137).

The findings of this study also highlight the importance of multisectoral governance and interinstitutional coordination in the success of child nutrition policies. Regional evidence suggests that countries achieving substantial reductions in child undernutrition have implemented integrated strategies combining social protection interventions, health system strengthening, food security, and social development policies (108, 138). In Peru, the Juntos conditional cash transfer program has been associated with significant reductions in the prevalence of child stunting over the past decade, within the context of a national multisectoral strategy to combat chronic undernutrition (126, 139). These experiences reinforce the importance of a comprehensive approach to addressing the structural determinants of malnutrition.

Despite the progress observed in several countries in the region, the data highlight persistent social and territorial inequalities in the distribution of child undernutrition. Numerous studies show that undernutrition remains more prevalent in rural populations, Indigenous communities, and households experiencing multidimensional poverty (10, 107). These inequalities reflect longstanding gaps in access to basic services such as safe water, sanitation, education, and health care. Consequently, interventions that fail to address these structural determinants may have limited or unequal impacts across different population groups (140).

Child undernutrition is a complex process reproduced across three interconnected domains: the general (economic, political, and cultural structures), the particular (the living conditions of social groups and their territories), and the singular (individual lifestyles and biological conditions). This perspective not only broadens the explanatory framework but also challenges interventions that attempt to address structural problems solely at the individual level (141).

### 4.1 Public health recommendations resource identification initiative

It is essential to consolidate sustained multisectoral strategies that integrate health, social protection, education, water, and sanitation. Regional evidence demonstrates that isolated interventions yield limited effects, whereas countries that have achieved greater progress (such as Peru and Brazil) have implemented integrated policies combining cash transfers, health services, and food security measures. In this regard, strengthening interinstitutional coordination mechanisms, ensuring stable financing, and establishing robust monitoring and evaluation systems are recommended to adapt interventions to specific contextual conditions.

Interventions during the first 1,000 days of life should be prioritized, including the strengthening of prenatal care, the promotion of breastfeeding, and appropriate complementary feeding. These actions should be accompanied by culturally adapted community-based interventions, as evidenced by local experiences (for example, the promotion of protein-rich foods such as eggs in Ecuador), which have proven effective when aligned with sociocultural contexts.

It is also crucial to expand and optimize nutrition-specific interventions, particularly food supplementation and fortification. Several countries have successfully implemented the fortification of staple foods with micronutrients (including iron, folic acid, vitamin A, and zinc), achieving significant improvements in child nutritional status. Examples include wheat flour fortification in Guatemala and the distribution of fortified foods in Chile. In this context, strengthening large-scale fortification programs with essential micronutrients, particularly zinc, is recommended, alongside ensuring appropriate regulation, coverage, and quality monitoring.

Furthermore, social protection programs, especially conditional cash transfers, should be strengthened and refined. Evidence indicates that these programs im-prove intermediate determinants such as access to food, utilization of health services, and childcare practices, although their impact on anthropometric indicators may vary. Therefore, these strategies should be complemented with interventions in health, nutrition, and education to maximize their effectiveness.

Strengthening school feeding programs and nutrition education is also recommended, ensuring the nutritional quality of food, program continuity, and effective linkage with health services. These programs have proven to be effective platforms for improving dietary intake and promoting healthy behaviors, particularly among vulnerable populations.

Finally, addressing the structural determinants of undernutrition is imperative, including poverty, inequality, access to safe water and sanitation, and territorial and ethnic disparities. Public policies should prioritize vulnerable populations (such as rural, Indigenous, and Afro-descendant communities) through equity- and rights-based approaches.

### 4.2 Limitations

Despite these efforts, important gaps in the available evidence across the region have been identified. Although a considerable number of studies evaluate large-scale social programs, there remains a limited body of research that examines in depth the processes of implementation, governance mechanisms, and contextual factors that influence the success or failure of interventions. As a scoping review, this study mapped the extent of the available evidence without conducting formal critical appraisal or quantitative synthesis; consequently, the heterogeneity of study designs and the potential for publication bias preclude causal inferences, and the mixed effects reported should be interpreted as a descriptive characterization of the evidence base rather than a weighted estimate of effectiveness.

Furthermore, there is a marked geographic evidence gap across the region, as well as a lack of comparative analyses across countries and between different policy types. While larger nations such as Brazil, Mexico, and Peru are extensively evaluated, several Latin America and the Caribbean countries, notably Costa Rica, Uruguay, and several Small Island Developing States in the Caribbean, are absent from the published policy evaluation literature. Expanding research coverage in underrepresented countries, strengthening monitoring and evaluation systems, promoting longitudinal studies, and advancing research that integrates both quantitative and qualitative approaches are essential to establish a comprehensive regional narrative and generate high-quality evidence capable of informing future public policies aimed at reducing child undernutrition in Latin America and the Caribbean through intergovernmental and interdisciplinary collaboration.

## 5 Conclusions

Child undernutrition in Latin America and the Caribbean constitutes a complex and multidimensional problem, closely linked to social, economic, and structural determinants. Evidence indicates that, although the region has made progress toward more comprehensive approaches that combine nutrition-specific and nutrition-sensitive interventions, outcomes remain heterogeneous and, in many cases, insufficient to achieve sustained reductions in indicators such as stunting. Therefore, the effective reduction of child undernutrition requires strengthening integrated, sustainable, and evidence-based public policies, with an emphasis on equity, multisectoral governance, and early intervention. To make future policy efforts actionable, research must address key evidence gaps, specifically regarding implementation science and governance mechanisms, long-term cost-effectiveness, programmatic impacts on the double burden of malnutrition, and geographic disparities in evidence across the region. Only through the articulation of strategies that simultaneously address both immediate and structural causes will it be possible to break the intergenerational cycle of undernutrition and advance toward more equitable development in the region.

## Data Availability

The original contributions presented in the study are included in the article/Supplementary material, further inquiries can be directed to the corresponding author.
